# Malaria transmission in landscapes with varying deforestation levels and timelines in the Amazon: a longitudinal spatiotemporal study

**DOI:** 10.1038/s41598-021-85890-3

**Published:** 2021-03-19

**Authors:** Gabriel Z. Laporta, Roberto C. Ilacqua, Eduardo S. Bergo, Leonardo S. M. Chaves, Sheila R. Rodovalho, Gilberto G. Moresco, Elder A. G. Figueira, Eduardo Massad, Tatiane M. P. de Oliveira, Sara A. Bickersmith, Jan E. Conn, Maria Anice M. Sallum

**Affiliations:** 1 Setor de Pós-Graduação, Pesquisa e Inovação, Centro Universitário Saúde ABC (FMABC), Fundação ABC, Santo André, SP Brazil; 2 Superintendência de Controle de Endemias (SUCEN), Secretaria de Estado da Saúde de São Paulo, Araraquara, SP Brazil; 3 Departamento de Epidemiologia, Faculdade de Saúde Pública, Universidade de São Paulo (FSP-USP), São Paulo, SP Brazil; 4 Unidade Técnica de Doenças Transmissíveis e Análise de Situação em Saúde, Pan American Health Organization (PAHO/WHO), Brasília, DF Brazil; 5 Coordenação-Geral de Vigilância de Zoonoses e Doenças de Transmissão Vetorial, Secretaria de Vigilância em Saúde, Ministério da Saúde (MS), Brasília, DF Brazil; 6 Fundação de Vigilância em Saúde do Amazonas, Manaus, AM Brazil; 7 Escola de Matemática Aplicada, Fundação Getúlio Vargas, Rio de Janeiro, RJ Brazil; 8 New York State Department of Health, The Wadsworth Center, Slingerlands, NY USA; 9 Department of Biomedical Sciences, School of Public Health, State University of New York, Albany, NY USA

**Keywords:** Epidemiology, Malaria

## Abstract

The relationship between deforestation and malaria is a spatiotemporal process of variation in *Plasmodium* incidence in human-dominated Amazonian rural environments. The present study aimed to assess the underlying mechanisms of malarial exposure risk at a fine scale in 5-km^2^ sites across the Brazilian Amazon, using field-collected data with a longitudinal spatiotemporally structured approach. Anopheline mosquitoes were sampled from 80 sites to investigate the *Plasmodium* infection rate in mosquito communities and to estimate the malaria exposure risk in rural landscapes. The remaining amount of forest cover (accumulated deforestation) and the deforestation timeline were estimated in each site to represent the main parameters of both the frontier malaria hypothesis and an alternate scenario, the deforestation-malaria hypothesis, proposed herein. The maximum frequency of pathogenic sites occurred at the intermediate forest cover level (50% of accumulated deforestation) at two temporal deforestation peaks, e.g., 10 and 35 years after the beginning of the organization of a settlement. The incidence density of infected anophelines in sites where the original forest cover decreased by more than 50% in the first 25 years of settlement development was at least twice as high as the incidence density calculated for the other sites studied (adjusted incidence density ratio = 2.25; 95% CI, 1.38–3.68; *p* = 0.001). The results of this study support the frontier malaria as a unifying hypothesis for explaining malaria emergence and for designing specific control interventions in the Brazilian Amazon.

## Introduction

Increased deforestation of tropical forests coupled with the emergence of new malaria-endemic regions is among the greatest obstacles to environmental sustainability, socioeconomic development and maintenance of the success of public health programs^1–3^. Despite major progress in malaria control after decades of intensive interventions, Brazil had ~ 194,512 cases of malaria in 2018, and 75% of the estimated 1 million annual malaria cases in Latin America occurred in Brazil and Venezuela^4^. In 2018 more than 40 million people in Brazil were at risk of contracting malaria and were threatened by the increased incidence of the disease compared to in 2015^4^. The highest malaria burden and incidence occur in rural communities and mining settlements across the Amazon basin. In these areas, malaria is primarily associated with deforestation, ecological changes, and intensive human movement linked to an ongoing process of land occupation^5–7^.

Deforestation and forest fragmentation favor the spread of major malaria vectors such as *Anopheles bancroftii, Anopheles farauti, Anopheles funestus* s.l*., Anopheles gambiae* s.l., and *Anopheles subpictus* in different geographical areas with endemic malaria transmission^8^. Across the Amazon basin, *Nyssorhynchus darlingi* is widely recognized as the primary vector of *Plasmodium* spp. in human-dominated landscapes impacted by deforestation. In such areas, the intensification of *Plasmodium* transmission and increased malaria incidence occur at the interface of natural and human-dominated environments^9^. Activities related to forest cover clearing, soil break-up, and crop cultivation can multiply the number of these interface environments in the landscape^10^. In addition, increased abundance of anopheline breeding sites and poor housing can coexist^11^, and together they favor the increase in vector species density, human-mosquito contact rate, and higher probability of infectious bites^9,12^. Anopheline mosquitoes, particularly *Ny. darlingi*^13^, have shown the ability to adapt rapidly to the process of forest clearing and fragmentation in the Amazonian agricultural frontier^14,15^. The primary drivers of increased malaria risk in endemic countries, including Brazil, are deforestation^16^, changes in mosquito communities, biodiversity losses linked to agriculture^17,18^, infrastructure development projects, such as hydropower plants^19^, fish-farming, mining activities, climate change, unplanned urbanization^20,21^, and the invasion of indigenous lands for illegal logging and mining^22,23^. Taken together, these land use alterations can likely increase the vectorial capacity of *Ny. darlingi*, leading to significantly higher malaria risk^15^.

Previous successful conservation policies such as those related to the expansion of indigenous lands and protected inhabited (indigenous lands, extractive reserves, and national forests) and uninhabited areas have made Brazil a global leader in environmental protection and indigenous rights advocacy^24^. To protect and promote conservation of the Amazon tropical rainforest, Brazil developed an advanced environment surveillance system, using satellite-based maps, to monitor fire and deforestation^25^. Unfortunately, since 2019, the political base that previously supported the protection of areas of frontier expansion has eroded and Brazil’s formerly exemplary environmental governance has been dismantled to exploit natural commodities. The harmful and damaging consequences, including for public health, of the native forest loss have not been considered. Former forest protection has been replaced by erratic and uncontrolled activities that have increased the exploitation of commodities, causing additional forest clearing across the Brazilian Amazon frontier^26^. From August/2019 to July/2020, deforestation increased by up to 9.5% compared with 10,129 km^2^ and 47% compared with 7536 km^2^ in the same periods in 2018–2019 and 2017–2018, reaching 11,088 thousand km^2^ based on historical data collected from the Amazonian forest monitoring system^27^. Among the consequences caused by the increased rate of deforestation^24,26^, the exponential augmentation in malaria incidence^1,3^ will ultimately cause widespread human illness, suffering and economic losses^18,28^.

**Figure 1:**
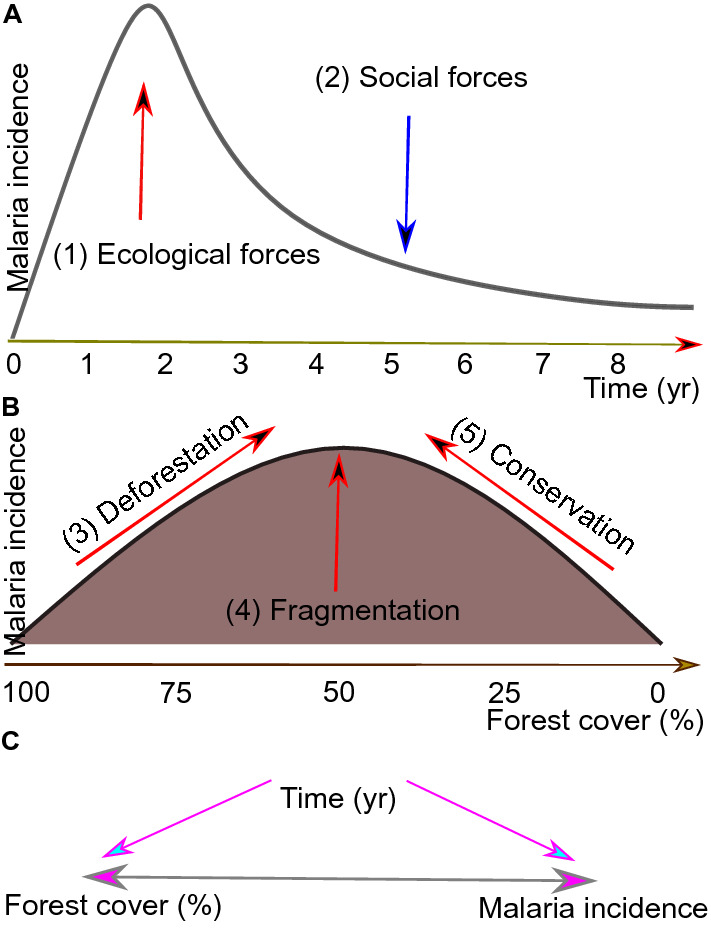
Theoretical background. (**A**) Malaria incidence increases in the early years of a human settlement in the Amazon, reaches a peak and then declines. This pattern is referred to as the FMH. A generalization of this pattern reveals that the most common curve of the malaria incidence distribution over time in a landscape impacted by changes in land use is convex. (1) Ecological forces: environmental driving forces in the high-risk scenario of malaria transmission at the beginning of colonization. (2) Social forces: a counterbalancing effect due to malaria commodities and life improvements that decrease malaria incidence over the long term. (**B**) malaria incidence increases with (3) deforestation: loss of forest cover from 100 to 50%. The underlying landscape mechanism linked to the DMH is (4) fragmentation: an increase in the frequency of forest fringe in the landscape that is the preferred habitat of *Ny*. *darlingi*. (5) Forest conservation in protected areas can result in a high frequency of humans at the forest fringe, increasing contact with *Ny*. *darlingi*. (**C**) the association between forest cover (or accumulated deforestation) and malaria incidence is affected by time, which is a factor that can modulate both variables in different ways. The forest cover-malaria incidence relationship can be bidirectional^5,8,16,17^.

A distinctive pattern of temporal and spatial change in malaria incidence has been described across frontier zones in the Amazon. This pattern is primarily associated with land occupation for the expansion of agribusiness and cattle ranches^10,17,29^. In the first stage of land occupation, newly deforested areas are heavily impacted by rapid intensification in malaria transmission. This is followed by stabilization of the disease occurrence, and after several years malaria incidence decreases^10,17^. The underlying mechanisms associated with those consecutive transmission stages are: (1) ecological factors, e.g., deforestation and biodiversity loss favor increase in malaria vector abundance and *Plasmodium* infection rate; and (2) social factors, e.g., improved human dwellings and better access to malaria commodities lead to a decrease in human and mosquito infection rates, human-mosquito contact rate and parasite transmission^17^. The equilibrium between these mechanisms of *Plasmodium* transmission is reflected in the pattern of convex curves of estimated malaria incidence over time (Fig. 1A)^10,17^. This is the expected transmission scenario associated with the frontier malaria hypothesis (FMH)^10,17,29^.

There is spatial–temporal variation in the distribution of malaria incidence, with both mosquito vector populations and transmission foci clustered in relatively small areas (~ 5 km^2^)^14,15,30–32^. The dominant malaria vector in the Amazon basin, *Ny*. *darlingi*, benefits from recently deforested landscapes intermixed with human-modified habitats^33–39^. This process of forest fragmentation creates the forest fringe effect in which malaria incidence increases due to expanded host-vector contact rates when human dwellings encroach on or are very near the forest edge^40–43^. The pattern of malaria incidence and accumulated deforestation at a fine scale is represented by a unimodal curve (Fig. 1B), associated with the deforestation-malaria hypothesis (DMH)^6,44^.

Time can confound the association between forest cover and malaria incidence (Fig. 1C). Local mechanisms of malaria risk and transmission intensity encompass: (1) deforestation is a temporal process^17^, thus, the older an anthropogenic landscape is, the more deforested and degraded it will be, and both vector abundance and malaria risk are likely to decrease^44^; (2) social determinants such as income, wealth, health services, education, and occupation improve over time, concomitant with a decline in malaria incidence^3,17^; and (3) the relationship between forest cover and malaria incidence is bidirectional; thus, increased malaria incidence decreases deforestation^2^, at least in the short term (Fig. 1C).

While investigations using wide spatial and temporal scales are important for depicting “big picture” scenarios^2,6,42^, field and community-based data collection provide detailed information at the local landscape scale of *Plasmodium* transmission and malaria incidence^15^. The novelty of our study is that it focuses on the local mechanisms of transmission, such as the fine structures of forest clearance and human exposure to *Ny*. *darlingi*. Such a scale allows for rigorous testing of the FMH^10,17,29^ and the DMH^9,33,42^. Furthermore, our conceptual framework of accessing *Ny*. *darlingi* in human settlements is original—we performed a pioneering longitudinal study that uses a spatial–temporal approach for Amazonian malaria landscapes. The goal of the study was to assess the effects of accumulated deforestation and the deforestation timeline on the distribution of *Plasmodium*-infected anophelines seeking human hosts in fine-scale (5 km^2^) sites with reported malaria transmission across the Amazonian states. Testing this allowed for an evaluation of the observed data of malarial exposure risk in Amazonian sites considering the underlying mechanisms ascribed to the FMH and the DMH.

## Results

A total of 21,242 anopheline specimens belonging to 37 species was collected from 80 5-km^2^ sites in 12 municipalities in four Brazilian Amazon states between 2015 and 2017 (Table 1). The primary anopheline vector (*Ny*. *darlingi*) was the most abundant (*n* = 18,353; 86%) among anophelines collected (Table 1). The overall *Plasmodium* infection rate from this vector was 1.04% (191/18,353). However, this rate varied among municipalities and was as high as 3.7% (39/1045) in Cruzeiro do Sul and as low as 0.06% (4/6811) in Presidente Figueiredo (Table 1). The *Plasmodium* infection rate from the other anophelines overall was 0.4% (12/2889) and that of the other anopheline species previously implicated as local malarial vectors found infected was 0.8% (12/1549).

**Table 1:** Numbers of collected specimens tested for the presence of *Plasmodium* according to anopheline species, infection status, and municipality of origin.

Municipality	Anophelinae species collected and infected by *Plasmodium*^1^							Anophelinae species collected and noninfected by *Plasmodium* N
	dar N (n infected)	ben N (n infected)	konB N (n infected)	oswA N (n infected)	per N (n infected)	ran N (n infected)	tri N (n infected)	
Acrelândia	270	3	500 (3)^**#**^,(1)*	1	0	116 (1)^**&**^	29	626
Cruzeiro do Sul	1045 (4)^**#**^,(35)^**&**^	3	19	0	2	6	17	41
Guajará	558 (5)^**#**^,(4)^**&**^	0	0	2	19	1	2	23
Humaitá	1004 (7)^**&**^	0	0	1	20	0	3	126
Itacoatiara	140 (1)^**&**^	0	0	0	12 (1)^**&**^	0	0	11
Lábrea	2122 (26)^**#**^,(8)^**&**^	0	1	7	0	0	0	116
Machadinho D’Oeste	1187 (3)^**#**^,(29)^**&**^	3	0	15 (1)^**&**^	0	2	351	30
Mâncio Lima	933 (5)^**#**^,(2)^**&**^,(1)*	2	10	1	1	0	2	39
Pacajá	42	18 (2)^**&**^	0	0	0	0	43	65
Presidente Figueiredo	6811 (4)^**&**^	0	0	2	0	0	133	208
Rodrigues Alves	1250 (7)^**#**^,(8)^**&**^	5	2	0	12	1	182 (3)^**#**^	48
São Gabriel da Cachoeira	2991 (5)^**#**^,(36)^**&**^	0	0	0	0	0	0	7

^1^dar, *Ny*. *darlingi*; ben, *Ny*. *benarrochi* s.l.; konB, *Ny*. *konderi* B; oswA, *Ny*. *oswaldoi* A; per, *An*. *peryassui*; ran, *Ny*. *rangeli*; tri, *Ny*. *triannulatus.*

^2^*Ny*. *albitarsis*, *Ny*. *arthuri*, *Ny*. *braziliensis*, *An*. *costai*, *Ny*. *deaneorum*, *Ny*. *dunhami*, *An*. *forattinii*, *Ny*. *goeldii*, *Ny*. *marajoara*, *An*. *mattogrossensis*, *An*. *minor*, *An*. *fluminensis*, *An*. *malefactor*, *An*. *punctimacula*, *Ke*. *neivai*, *St*. *nimbus*, *Ny*. *nuneztovari*, *Ny*. *oryzalimnetes*, *An*. *shannoni*, *St*. *thomasi*, *Chagasia fajardi*, and other unidentified anopheline species^14^.

^**#**^*Plasmodium falciparum*.

^&^*Plasmodium vivax*.

*Mixed infection (*Plasmodium vivax* and *Plasmodium falciparum*).

From the twelve municipalities studied, all had at least one site with the primary anopheline vector (*Ny*. *darlingi*) and/or local malarial vectors infected with *Plasmodium falciparum* and/or *Plasmodium vivax* (Tables 1, 2). Only two municipalities (Acrelândia and Pacajá) had no infected *Ny*. *darlingi*, but instead had exclusively local malarial vectors (Table 1). The occurrence of positive sites for *Plasmodium* presence varied among municipalities (Table 2). Only one site (1/6; 17%) was positive in Pacajá, whereas all sites (7/7; 100%) were positive in São Gabriel da Cachoeira (Table 2). Overall, just over half of the sites (41/80; 51.25%) contained *Plasmodium* (Table 2). Accumulated deforestation, estimated as the inverse of forest cover, varied from 9.9 to 89% (mean = 50.2, sd = 18.9), whereas the deforestation timeline, estimated as the number of years since the beginning of the organization of the settlement, varied from 0 to 45 years (mean = 26.2, sd = 13.9) among these 41 sites (Table 2). Considering only the infected anophelines, the proportion of infected *Ny*. *darlingi* with *Plasmodium vivax* was 70.7% (135/191) and the proportion of those with *Plasmodium falciparum* was 29.3% (56/191), while the proportion of local malarial vectors with *P*. *vivax* or *P*. *falciparum* was 50% (6/12) (Table 2). These 41 sites were included in the statistical modeling and the hypothesis testing analyses, as detailed in the Data Analysis section below.

**Table 2:** Number of infected anophelines, accumulated deforestation and deforestation timeline by site, municipality, and state, 2015–2017.

State	Municipality	Site (5-km^2^)	AD^2^ (%)	DT^2^ (Yr.)	Number of infected anophelines			
					*Ny*. *darlingi*		Secondary Vectors	
					Viv N	Falc N	Viv N (sp.^1^)	Falc N (sp.^1^)
AC	Acrelândia	L2 Francisco	25.93	4	0	0	1 (konB)*	1 (konB)*
AC	Acrelândia	L2 Marinalva	29.87	9	0	0	0	3 (konB)
AC	Acrelândia	L3 Porto Dias	29.87	9	0	0	1 (ran)	0
AC	Cruzeiro do Sul	L1 Saboeiro	57.74	40	1	0	0	0
AC	Cruzeiro do Sul	L2 Cohab	84.09	40	31	0	0	0
AC	Cruzeiro do Sul	L6 PDS Jamil Jere	56.3	31	1	0	0	0
AC	Cruzeiro do Sul	L7 Ramal Caraca	43.86	32	2	4	0	0
AM	Guajará	L1 Ig. Grande	74.29	45	0	2	0	0
AM	Guajará	L2 Vila Gama	51.17	45	2	0	0	0
AM	Guajará	L3 Ramal do G	38.8	13	1	0	0	0
AM	Guajará	L4 Badejo Meio	70.78	45	1	3	0	0
AM	Humaitá	L1 Cristolandia	36.45	32	2	0	0	0
AM	Humaitá	L3 Realidade	66.36	32	5	0	0	0
AM	Itacoatiara	L1 Novo Reman	32.16	5	1	0	0	0
AM	Itacoatiara	L6 Novo Reman	40.18	21	0	0	1 (per)	0
AM	Lábrea	L1 Umari BA	30.1	18	1	0	0	0
AM	Lábrea	L2 Umari BA	41.66	18	1	8	0	0
AM	Lábrea	L3 Pacia	9.86	0	6	3	0	0
AM	Lábrea	L4 Pacia	22.81	15	0	14	0	0
AM	Lábrea	L5 BR 230 km	88.95	30	0	1	0	0
RO	Machadinho D’Oeste	L1 Galo Velho	68.76	10	6	0	0	0
RO	Machadinho D’Oeste	L2 Galo Velho	69.81	10	21	0	1 (oswA)	0
RO	Machadinho D’Oeste	L3 Galo Velho	46.56	5	1	3	0	0
RO	Machadinho D’Oeste	L4 Galo Velho	76.68	10	1	0	0	0
AC	Mâncio Lima	L1 Guarani	49.33	40	2*	2*	0	0
AC	Mâncio Lima	L2 Guarani	54.33	40	1	1	0	0
AC	Mâncio Lima	L5 Pentecoste	32.54	35	0	1	0	0
AC	Mâncio Lima	L6 Pentecoste	24.24	14	0	2	0	0
PA	Pacajá	L6 Cururui	34.97	11	0	0	2 (ben)	0
AM	Presidente Figueiredo	L1 Jerusalem	60.61	42	4	0	0	0
AC	Rodrigues Alves	L1 Sitio Ie	47.41	17	4	2	0	3 (tri)
AC	Rodrigues Alves	L2 Agrovila	61.75	42	3	1	0	0
AC	Rodrigues Alves	L4 Ramal Buriti	82.98	42	0	2	0	0
AC	Rodrigues Alves	L6 Faz Sr J	53.14	42	1	2	0	0
AM	São Gabriel Cachoeira	L1 Com SA	46.94	33	4	0	0	0
AM	São Gabriel Cachoeira	L2 Com Ita Mir	55.74	33	1	0	0	0
AM	São Gabriel Cachoeira	L3 Sitio Bene	42.48	33	14	3	0	0
AM	São Gabriel Cachoeira	L4 T Montalvo	77.75	33	11	2	0	0
AM	São Gabriel Cachoeira	L5 M Quirino	60.28	33	1	0	0	0
AM	São Gabriel Cachoeira	L6 Com Boa Esp	46.18	33	3	0	0	0
AM	São Gabriel Cachoeira	L7 Sitio PG	33.82	33	2	0	0	0

^**1**^ben, *Ny*. *benarrochi* s.l.; konB, *Ny*. konderi B; oswA, *Ny*. *oswaldoi* A; per, *An*. *peryassui*; ran, *Ny*. *rangeli*; tri, *Ny*. *triannulatus.*

^**2**^site features: AD, accumulated deforestation (%); DT, deforestation timeline (age of settlement in years).

*one mixed infection (*P*. *vivax* and *P*. *falciparum*).

**Figure 2:**
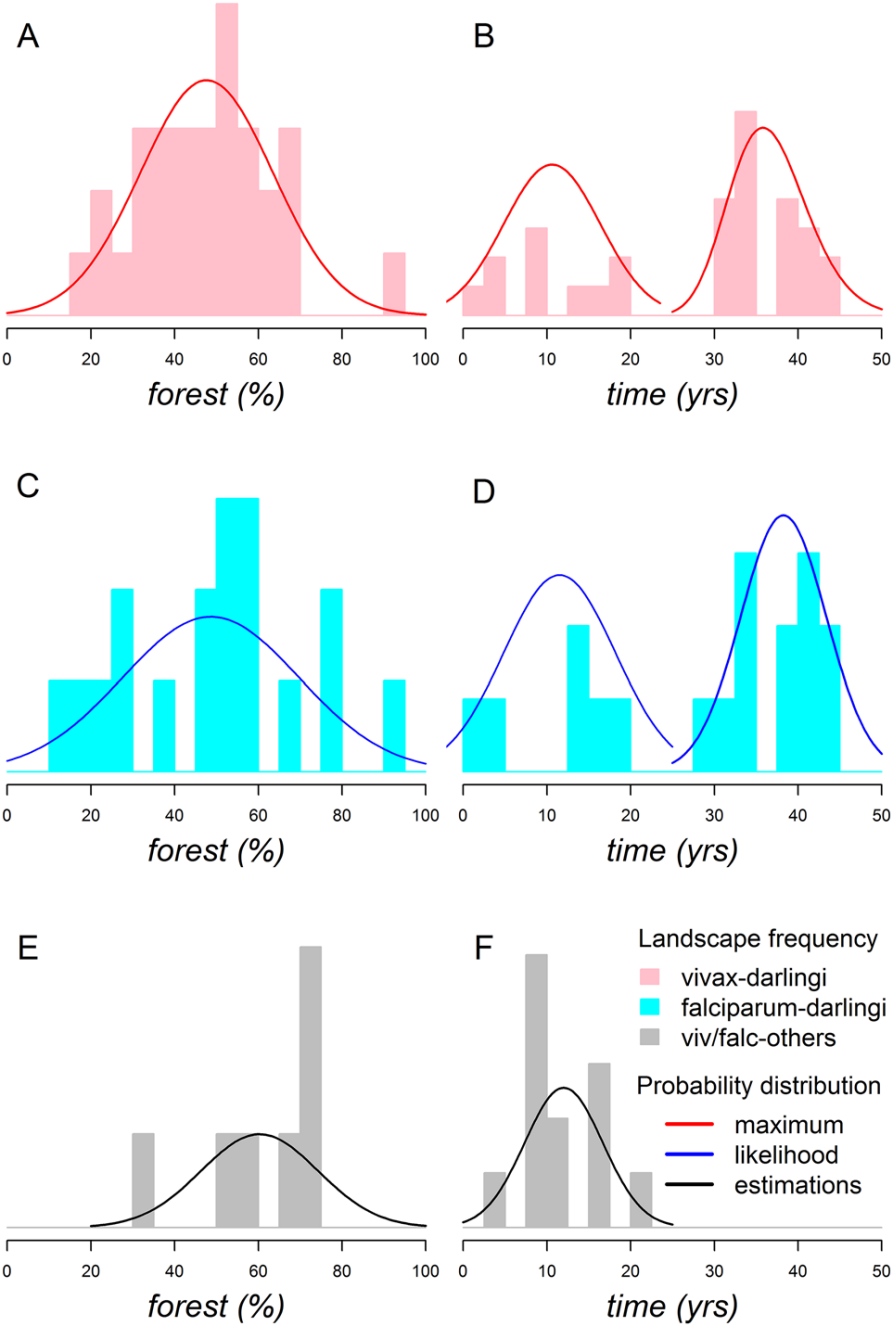
Site frequency of *Plasmodium*-infected anophelines along gradients of forest cover and time. (**A**) *P*. *vivax*-*Ny*. *darlingi* occurrence peaked in sites where the percentage of forest cover was intermediate (~ 50%). The maximum likelihood probability curve was estimated as a Gaussian distribution (mean = 47.64, sd = 15.86; *p* < 0.001). (**B**) *P*. *vivax*-*Ny*. *darlingi* occurrence showed two deforestation timeline peaks at (1) ~ 10 years (Gaussian distribution; mean = 10.6, sd = 5.77; *p* < 0.001) and (2) ~ 35 years (Log-normal distribution; meanlog = 3.59, sdlog = 0.13; *p* < 0.001) from the start of deforestation for the establishment of human settlements. The deforestation timeline distributions are significantly different (t = 12, df = 15, *p* < 0.001). (**C**) *P*. *falciparum*-*Ny*. *darlingi* occurrence was higher in sites with intermediate levels of forest cover (~ 50%) (Gaussian distribution; mean = 48.64, sd = 21.12; *p* < 0.001). (**D**) The deforestation timeline curves for the occurrence of infected mosquitoes were observed at (1) ~ 10 years (Gaussian distribution; mean = 11.5, sd = 6.65; *p* < 0.001) and (2) ~ 40 years (Gaussian distribution; mean = 38.25, sd = 5.1; *p* < 0.001). The deforestation timeline distributions were significantly different (t = 8, df = 8, *p* < 0.001). (**E**), *P*. *falciparum* and/or *P*. *vivax* occurrence in other Anophelinae species was higher in sites with forest cover of ≥ 60% (Gaussian distribution; mean = 60.28, sd = 14; *p* < 0.001). (**F**), A deforestation timeline peak at 12 years from the beginning of human occupation (Gaussian distribution; mean = 12, sd = 4.67; *p* < 0.001).

*Plasmodium vivax*-*Ny*. *darlingi* and *P*. *falciparum*-*Ny*. *darlingi* occurrence peaked in sites where the percentage of forest cover was intermediate with a maximum likelihood mean of 47.6% (Fig. 2A) and 48.6% (Fig. 2C), whereas *P*. *falciparum* and/or *P*. *vivax* occurrence in other anopheline species occurred in sites with a higher forest cover with a maximum likelihood mean of 60.3% (Fig. 2E). In considering the deforestation timeline only, two peaks were estimated by maximum likelihood. The first peak occurred 10–12 years after the beginning of the organization of a settlement in *Plasmodium vivax*-*Ny*. *darlingi* (Fig. 2B), *P*. *falciparum*-*Ny*. *darlingi* (Fig. 2D) or in local malarial vectors (Fig. 2F). The second peak occurred 36–38 years after the beginning of the organization of a settlement in *Plasmodium vivax*-*Ny*. *darlingi* (Fig. 2B) or *P*. *falciparum*-*Ny*. *darlingi* (Fig. 2D), when secondary malarial vectors were absent (Fig. 2F).

The next confirmatory analysis was run to estimate the incidence density ratio (IDR) of infected anophelines to the combined effects of accumulated deforestation and the deforestation timeline adjusted by control variables. The number of infected anophelines followed a Poisson distribution (rate ratio = 1.2, 95% CI = 0.5–3.9, *p* = 0.023), ranging from 1 to 31 females per site (mean = 4.8, sd = 6.3) (Table 2). The accumulated deforestation and deforestation timeline were strongly and positively correlated with each other (*r* = 0.52, t = 3.815, df = 39, *p* < 0.001) (Table 2). Sites with ≥ 50% forest cover were defined as preserved forest, otherwise as degraded; sites with ≤ 25-years of deforestation timeline were defined as new settlements, otherwise as old (Table 3). The adjusted IDR of new-degraded sites was 2.25 (95% CI, 1.38–3.68; *p* = 0.001), whereas the adjusted IDR of new-preserved sites was 0.42 (95% CI, 0.25–0.69; *p* < 0.001) and those of old-degraded or old-preserved sites were 0.49 (*p* < 0.02) (Table 3). This means that new-degraded sites had the highest malarial exposure risk, and the other two variable combinations tested had the lowest (Table 3). Therefore, new-degraded sites represent the risk scenarios, whereas new-preserved, old-preserved, and old-degraded sites represent the protection scenarios (Table 4). This overall pattern of malarial exposure risk was mainly caused by the effect from *P*. *vivax*-*Ny*.*darlingi* (Table 4). Considering only the effect from *P*. *falciparum*-infected *Ny*. *darlingi*, the malarial exposure risk was higher in new-preserved sites (Table 4). Local malarial vectors also have a significant effect on malaria transmission in new-preserved sites (Table 4).

**Table 3:** *Poisson* generalized linear models of mean numbers of infected anophelines as a function of the combined effects of accumulated deforestation and the deforestation timeline adjusted by control variables.

Landscape scenario (Combined effects)^**1**^	Adjusted-IDR^**2**^ (95% CI)	*p* (Wald’s test)
New-preserved sites (≥ 50% forest cover and ≤ 25 years deforestation timeline)	0.42 (0.25–0.69)	< 0.001
Old-preserved sites	0.49 (0.27–0.86)	0.014
Old-degraded sites	0.49 (0.28–0.85)	0.011
New-degraded sites	2.25 (1.38–3.68)	0.001

^**1**^Preserved and new sites (14/41 = 34.2%), preserved and old sites (8/41 = 19.5%), degraded and new sites (3/41 = 7.3%), and degraded and old sites (16/41 = 39%).

^**2**^The adjusted incidence density ratio of infected anophelines between the new-degraded landscape scenario and the three other landscape scenarios adjusted by the number of collectors (3 or 4) and the period of collection (12 h or 6 h).

**Table 4:** Risk/protection landscape scenarios based on incidence density ratio of infected anophelines and the combined effects of accumulated deforestation and the deforestation timeline adjusted by control variables.

Landscape scenarios (combined effects)^**1**^	Adjusted IDR of infected anophelines^6^		
	vivax-*Ny. darlingi*	falciparum-*Ny. darlingi*	*Plasmodium*-local malarial vectors
New-preserved sites (≥ 50% forest cover and ≤ 25 years deforestation timeline)	Protection^**2**^	Risk^**3**^	Risk^**3**^
Old-preserved sites	Protection^**2**^	No effect^**4**^	-^**5**^
Old-degraded sites	Protection^**2**^	No effect^**4**^	-^**5**^
New-degraded sites	Risk^**3**^	-^**5**^	No effect^**4**^

^1^preserved and new sites (14/41 = 34.2%), preserved and old sites (8/41 = 19.5%), degraded and new sites (3/41 = 7.3%), and degraded and old sites (16/41 = 39%).

^2^Adjusted IDR of infected anophelines < 1, *p* < 0.05, and baseline = new-degraded sites.

^3^Adjusted IDR of infected anophelines > 1, *p* < 0.05.

^4^Adjusted IDR of infected anophelines = 1, and baseline = new-preserved sites.

^5^No infected anopheline found.

^6^Incidence density ratio (IDR) was adjusted by the number of collectors (3 or 4) and the period of collection (12 h or 6 h).

## Discussion

The underlying mechanisms of the FMH and the DMH are based on the patterns of human exposure risk across agricultural frontiers in the Brazilian Amazon. The FMH^10,17,29^, currently the main theoretical model for predicting both malaria emergence and decline in Amazonian agricultural settlements, was tested by considering one of its predictors, i.e., the deforestation timeline. The deforestation timeline of a given site represents the natural-history succession of *Plasmodium* transmission in local settler communities. In Fig. 2, four main stages were observed: (1) during the first 10 years of the initiation of a settlement, malaria incidence surges; (2) after the first malaria transmission peak that occurs at approximately 10–15 years, transmission is reduced; (3) after consolidation of the settlement, malaria occurrence declines from 15 to 30 years on the timeline; and (4) a second peak can occur after 30 years. The deforestation timeline was challenged by an alternative explanation: that the proportion of remaining forest cover in a site, independent of the deforestation timeline, is a better predictor for malaria occurrence^6,31,44^. In Fig. 2, it was shown that the accumulated deforestation from 30 to 70% contained the highest risk of malaria transmission. This is related primarily to loss of forest cover leading to fragmentation and greater forest fringe frequency^7,8^. These features promote a higher abundance of habitats for *Ny*. *darlingi* and intensification of the contact rate among *Plasmodium*-infected hosts, competent vectors, and susceptible human hosts^9,20,40^. It is clear therefore that deforestation timeline and accumulated deforestation are important determinants for malaria risk in the Amazon basin. Considering that accumulated deforestation is the only underlying mechanism in the DMH, whereas both deforestation timeline and accumulated deforestation are underlying mechanisms in the FMH, we suggest that the obtained findings in here provide robust support for the validity, reliability, and inference power of the FMH.

**Figure 3:**
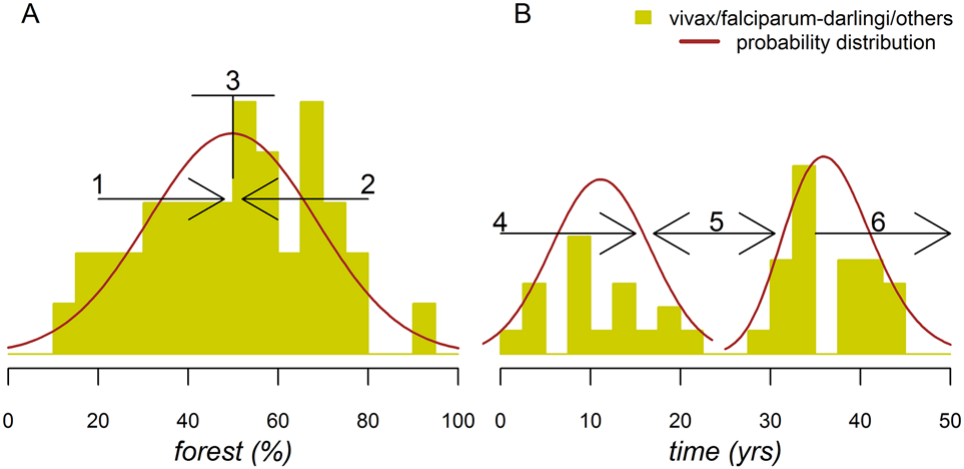
Pathogenic site frequency with forest cover and time. (**A**) Maximum frequency in sites having an intermediate percentage of forest cover (= 50%; Gaussian distribution; mean = 49.8, sd = 18.7; *p* < 0.001). (**B**) Bimodal presence with two deforestation timeline peaks, one peak at (1) 11 years (Gaussian distribution; mean = 11.1, sd = 5.5; *p* < 0.001) and a second peak at (2) 37 years after starting the deforestation process (log-normal distribution; meanlog = 3.6, sdlog = 0.13; *p* < 0.001). The two deforestation timeline distributions were significantly different (t = 15, df = 32, *p* < 0.001). Underlying mechanisms of malaria risk according to forest cover and time: 1, forest conservation^42^; 2, deforestation^33^; 3, the forest fringe hypothesis^9^ and the FMH^10,17^; 4, the FMH^10,17^; 5, malaria-deforestation bidirectional effects ^2^ and 6, a rebound in malaria transmission^2,10,17^.

Results of Fig. 2 were synthesized, summing up all vectors and malarial parasites according to the forest cover gradient (Fig. 3A) and the deforestation timeline (Fig. 3B). Overall, this shows that the maximum peak of pathogenicity occurs in sites with 50% forest cover (Fig. 3A). Our previous mathematical modeling revealed that high *Ny*. *darlingi* population abundance enables malaria transmission through elevated levels of human biting rates in these sites^15,45^. Establishment of rural settlements can contribute to malaria transmission in sites with high densities of anopheline vectors and a more stable human population, such as in landscapes with 50% forest cover^6,31,44^. Because *Ny*. *darlingi* proliferates in fragmented landscapes with intermediate forest cover levels (~ 50%), it is reasonable to suppose that malaria risk presents a unimodal pattern (Fig. 3A) along the gradient of forest cover^6,9,23,33–35,42,44,46^. The sites with 50% forest cover have (1) the highest pathogenic input because they have the highest levels of vector abundance and host-vector contact rate^5,40,41^; and (2) the lowest probability of a consolidated socioeconomic ecosystem to increase access to malaria commodities^1,10,15,17,20^.

Distinct from the FMH, our results showed two peaks of pathogenic site frequency per deforestation timeline, ~ 10 and 35 years after the beginning of a settlement (Fig. 3B). The gap between the peaks is likely due to a bidirectional effect, recently proposed using municipality-level data, in which deforestation triggers malaria incidence, which in turn decreases the intensity of deforestation^2^. The gap underscores the deceleration of malaria incidence after 10 years, possibly due to decreased deforestation levels, improved socioeconomic environment and better access to diagnosis and treatment^10,17^. Nevertheless, the pathogenicity of the site in terms of vulnerability and receptivity to *Plasmodium* transmission remains^1,41^, and is visualized as a second malaria peak 35 years after the beginnings of a settlement (Fig. 3B). This peak is associated with a second wave of colonization, expansion of local deforestation frontiers, increased human population resulting from the growth of families, and settler migration from other malaria-endemic areas into a newly colonized area for land occupation^40,41^. There are several possible reasons for the second peak: a malaria rebound in the same location, a late first emergence due to human mobility, the immigration of the malarial naïve population into the settlements, expansion of the settlement into neighboring forested areas, and (or) the presence of undetected asymptomatic *Plasmodium*-infected people in the community^40^.

The natural-history succession of *Plasmodium* transmission in a local settler community begins with *P*. *falciparum* transmitted by *Ny*. *darlingi*^41^ with the contribution of local vectors (Table 4) when this community with little or no access to health facilities starts a deforestation process in a preserved site with roughly 100% forest cover^20,35,41,46^. Deforestation increases the larval habitats of *Ny*. *darlingi*, increasing its abundance. This mosquito searches for human blood inside precariously-constructed housing or shelters, increasing malaria transmission^40^. The introduction of *P*. *vivax* complicates malaria control in this community because the combined incidence density of both *P*. *falciparum* and *P*. *vivax* transmitted by *Ny*. *darlingi* results in the highest malarial risk (seen in Tables 3, 4) attributed to human colonization in newly deforested and highly degraded sites. As the community becomes better organized and access to health infrastructure improves, the expanded surveillance and health care systems contribute to *P*. *falciparum* niche replacement by *P*. *vivax*^17,41,47^. This may lead to a stable or decreasing transmission with the long-term persistence of *P*. *vivax*^40,41^. Although a second malaria peak is possible in the same community with the participation of *P*. *falciparum* and *P*. *vivax* (Fig. 2), the incidence density of the second peak is lower in comparison with the first peak (Tables 3, 4). This further suggests that sites that have been occupied the longest, i.e., with more than 25 years since the beginning of the settlement, generally represent the lowest risk in comparison with newly occupied sites.

Local malarial vectors are ignored in the FMH or in the DMH. In almost 20% of the municipalities sampled, we did not detect infected *Ny*. *darlingi*, but found naturally-infected *Nyssorhynchus konderi* B and *Nyssorhynchus benarrochi* s.l. that we hypothesize are the local vectors (Table 1). In Acrelândia municipality, Acre state, *Ny*. *konderi* B outnumbered *Ny*. *darlingi* in the field collections. One rural settlement, Porto Dias, composed of a continually conserved forest^31^, has become an important malaria hotspot in Acrelândia. Our data suggest that the local *Plasmodium* vector is *Ny*. *konderi* B, which was infected with *P*. *falciparum* and *P*. *falciparum*/*P. vivax* at the forest edge. This result is supported by a previous study^48^ that identified *Ny*. *konderi* s.l. as a local malaria vector in another rural settlement in Acre. In addition, *Ny*. *benarrochi* s.l. is a local malaria vector in the Peruvian Amazon^49^ and in southern Colombia^50^; in the present study, *P. vivax* infected females were captured in the peridomestic habitat at Cururuí settlement, Pacajá municipality, Pará state.

We applied the concepts of landscape pathogenicity and landscape ecology of malaria in the Amazon in this study to enable a deeper understanding of the general land use dilemma in tropical rainforests^3,30^: (1) the conversion of the landscape’s abiotic and biotic factors is needed for the incorporation of valuable resources (i.e., economic goods and services) to society and local communities; but (2) such economic activities frequently connect vectors, hosts, and humans in the landscape and expose them to zoonotic pathogens^51,52^. The influence of deforestation and related human disturbances in Amazonian development projects is associated with the emergence and spread of several infectious and zoonotic diseases in addition to malaria^53,54^. It is only with the use of realistic and pragmatic control of deforestation of the Amazon tropical rainforest that biodiversity can be maintained and thus help in the protection of human health^53^. Brazil’s substantial responsibility in terms of environmental policies for Amazon forest conservation has been jeopardized by the Brazilian Government since 2019^24^. Forest cover losses in the tropical rainforests of Southeast Asia and Malaysia in recent decades may be linked to the origins of SARS-CoV-2; a similar phenomenon may be the basis of the COVID-19 pandemic^54^. From a global health perspective, Brazil’s lack of commitment to the preservation of the Amazon tropical rainforest will be reflected in long-term threats to human health^55^.

## Limitations

The findings of this study were based on an estimated 4200 h of field collections conducted in 80 sites across areas with active malaria transmission in the Brazilian Amazon. Despite our robust sample sizes that critically represent each of the four landscape categories (new-preserved, new-degraded, old-preserved, and old-degraded), a longitudinal study over several years of field collections per site and/or per municipality would have allowed us, in addition, to verify seasonal variation in each mosquito vector population, dynamics of transmission, and spatiotemporal variation of malaria incidence in communities in rural settlements.

## Concluding remarks

The null hypothesis that the FMH is the main predictor of the rise and fall of malaria transmission across Amazonian landscapes was tested against an alternative hypothesis, the DMH. It was found that malaria transmission not only rises in accordance with forest cover loss (deforestation), as stated in the DMH, but transmission varies because of the combined effects from accumulated deforestation and the deforestation timeline. Recently colonized sites that have been highly deforested comprise the highest transmission risk, whereas highly forested preserved sites or sites that have been occupied for a longer period (old settlements) present the lowest risk, in agreement with the FMH. Our findings demonstrate that the DMH further supports the FMH as a unifying concept for designing public policies for malaria elimination, and perhaps for the prevention of zoonotic diseases, in the Amazonian basin.

## Methods

### Study system and rationale

The highest malaria burden occurs in rural Amazonian Brazil^4–6^. Frontier agricultural settlements initiated in the late 1970s now number 3738, covering greater than 75 million ha^56^. Each agricultural settlement is a set of independent units, installed by the Instituto Nacional de Colonização e Reforma Agrária (INCRA), where there was a rural property that belonged to a single owner^56^. Each of these units is delivered by INCRA to a family that has no other means to acquire a rural property^56^. In 2018 alone, over 59,000 malaria cases occurred in these settlements^57^. These cases are clustered in those units most recently occupied, a pattern referred to as “frontier malaria”, leading to assumptions that malaria incidence can be directly predicted by settlement age^10,29^.

We reevaluate a pioneering study that first proposed the FMH^10,29^, by calculating forest cover (%) for the same site and years (Machadinho D’Oeste, Rondônia; 1984–1987, 1995, 2012) as their malaria incidence data. Both settlement age and cleared area have strong and qualitatively similar associations with malaria incidence; the correlation between settlement age and forest cover is *r* =  − 0.92. Thus, the depiction of FMH as a temporal progression due to settlement age cannot be distinguished from an alternative hypothesis of malaria incidence being driven by changes in forest cover. Our experiments are designed to evaluate the regional applicability of FMH by explicitly decoupling settlement age and forest cover effects by comparing different classes of forest cover in both new and old settlements^31^.

The 80 sampling units (sites) were located in settlements with active malaria transmission across the Brazilian Amazon in the states of Acre, Amazonas, Pará, and Rondônia. The selection of these sites for field collections was based on the pattern of frontier malaria regarding recently occupied land promoted by the INCRA as well as “old” occupied plots, some of which are still agriculture settlements, others having been modified into urban areas. We assumed that the accessible population of settlements was initiated within the scope of INCRA’s rural development policies. By assuming this we could use a space-for-time longitudinal approach^58^ in a temporal-environment-structured model in order to reconstruct the colonization process timeline.

### Criteria for choice of settlements and sites within settlements

Settlements were chosen according to the following eligibility criteria: (1) a high-to-moderate monthly parasite index (e.g., > 10 confirmed new cases of malaria per 1000 individuals) in the previous month of field collection; (2) the presence of malarial transmission (i.e., *Plasmodium*); (3) suitable aquatic habitats for *Ny*. *darlingi*, unprotective housing and/or personal anecdotes from locals about the occurrence of adult anophelines during the period of field collections; and (4) accessibility by road. Each site was georeferenced. Using GIS data from the Landsat Project (USGS) and Brazilian land cover maps (INPE), we defined 5-km^2^ blocks (i.e., sampling units) in each site centered on the geographic position of the peridomestic collection^30,31^. With Landsat 8-OLI satellite imagery from the year of collection, we identified the level of forest cover (range: 0–100%) over each 5-km^2^ block by applying a supervised classification algorithm in QGis v. 2.16.2 Nodebo^31^. Using all the available imagery databases from Landsat satellites (Landsat 1–8) from the 1970s on, we estimated the deforestation timeline from the beginning of settlement organization until the year of our field collection^31^. The approach used to estimate forest cover and deforestation timelines was previously published and can be found elsewhere^31^.

### Sampling strategy and design

**Figure 4:**
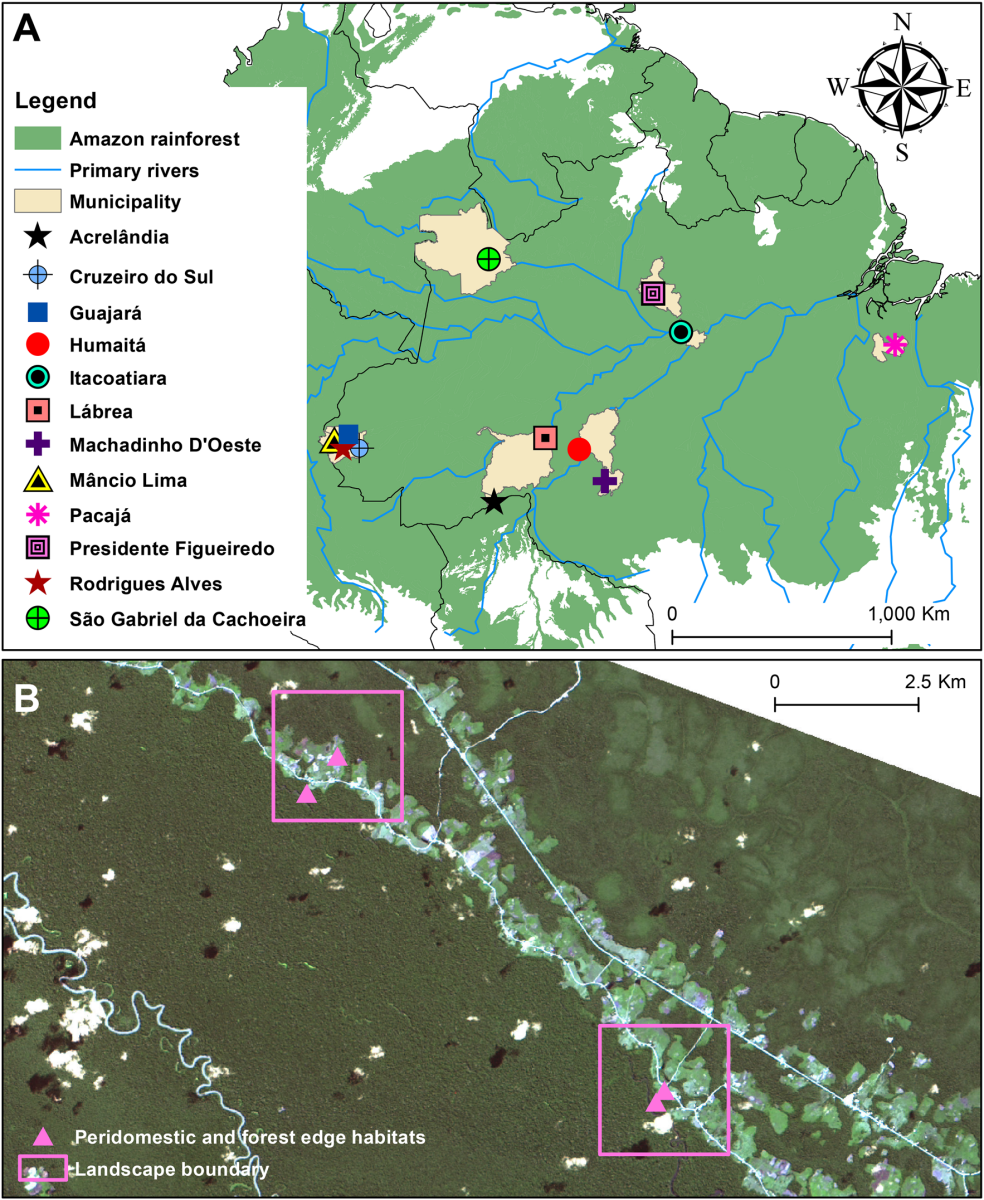
Malaria transmission in the Amazon rainforest. (**A**) Selected municipalities for estimating malarial exposure risk, forest cover and deforestation timeline at a fine scale (5 km^2^). These municipalities are characterized by a wet and a dry season with wet-dry transition months (mean annual rainfall > 2000 mm; mean annual temperature ~ 26 °C). The rural and peri-urban communities studied in these municipalities were selected as representative of areas with moderate or high malaria transmission. (**B**) Two 5-km^2^ sampling units are illustrated as examples. They are in the agricultural settlement of Pentecostes in the municipality of Mâncio Lima, Acre state. Inside each unit, the exact geographic points of mosquito collection in the peridomestic environment and on the forest edge are shown. Both sampling units were considered preserved sites and were selected for mosquito sampling during field collections performed in May 2015. As an example of how high the malarial exposure risk was, one of the collectors was infected by *P*. *falciparum* via a *Ny*. *darlingi* female in one of these sampling units. This RGB composite imagery was created by the first author (GZL) working with ArcGIS v. 10.3.1 and data from publicly available sources (USGS, Landsat Project, WWF, INPE).

Human-seeking mosquito collections were conducted in 13 field collections from 12 municipalities across the Brazilian Amazon (Fig. 4A). The levels of forest cover and deforestation timeline varied widely and randomly across the selected sites, although we strove to select equal numbers of degraded (0–29%), intermediate (30–49%), and preserved (≥ 50% forest cover) sites. Sites with similar forest cover statuses (e.g., degraded, intermediate, and preserved) were selected in the same way in each municipality. Six sites (e.g., 2 degraded, 2 intermediate, and 2 preserved) were sampled per municipality, except in Guajará and São Gabriel da Cachoeira in Amazonas state (Fig. 4A), where seven units were sampled in each. The 7th sites in Guajará and São Gabriel da Cachoeira had intermediate and preserved forest cover statuses, respectively, and they were selected to improve our sampling effort in these municipalities.

### Mosquito collections

Field collections were designed to address vector abundance and the prevalence of *Plasmodium* infection in the mosquito population and were conducted from January 2015 to November 2017. Thirteen collections of approximately 15 days each were made in twelve municipalities, as follows: (1) Acrelândia, Acre state (January 2015); (2) Cruzeiro do Sul, Acre state (April 2015); (3) Mâncio Lima, Acre state (May–June 2015); (4) Lábrea, Amazonas state (July–August 2015); (5) Acrelândia, Acre state (August 2015); (6) Machadinho D’Oeste, Rondônia state (October 2015); (7) Pacajá, Pará state (April 2016); (8) Humaitá, Amazonas state (July 2016); (9) Itacoatiara, Amazonas state (November 2016); (10) Rodrigues Alves, Acre state (June–July 2017); (11) Guajará, Amazonas state (July 2017); (12) Presidente Figueiredo, Amazonas state (August 2017); and (13) São Gabriel da Cachoeira, Amazonas state (November 2017). Mosquitoes were collected in a total of 80 sampling units (see each mosquito collection described in the Supplementary Material). Sampling consisted of 1 peridomestic and 1 forest edge collection conducted on the same night plus a second peridomestic collection the following night (Fig. 4B). Peridomestic collections occurred within ~ 5 m of each house, and forest edge collections took place within the forest edge nearest to the human dwellings (Fig. 4B). The linear distance between the peridomestic and forest edge collections ranged from 0.2 to 1 km (Fig. 4B). Mosquitoes were captured using (1) human landing catch (HLC) and (2) barrier screen sampling (BSS) in the peridomestic environment, and (3) Shannon traps (ST) at the forest edge (Fig. 4B). HLC (*n* = 80) and ST (*n* = 80) collections were conducted in parallel across all 80 sites (Supplementary Material); in contrast BSS was carried out for a subsample (*n* = 38) of the sampling units (Supplementary Material). The subsample comprised 1 degraded, 1 intermediate, and 1 preserved sampling unit per municipality, except in Mâncio Lima (*n* = 0), Itacoatiara (*n* = 4), Guajará (*n* = 5), São Gabriel da Cachoeira (*n* = 4), and Presidente Figueiredo (*n* = 4).

HLC and ST collections were carried out from 18:00 to 0:00 h or from 18:00 to 06:00 h. The number of collectors ranged from one to three individuals, depending on their availability during each field collection period. The lack of a standardized sampling plan was compensated for by the use of the number of collectors and the period of collections as control variables for adjusting the incidence density ratio (see “Data analysis” section). BSS occurred from 18:00 to 22:00 h, except during two collection events that occurred from 18:00 to 21:00 h because of adverse weather. Every hour, captured female mosquitoes were euthanized with ethyl acetate (C_4_H_8_O_2_) vapors in the field and stored in silica gel separated by date, location, house and hour of collection. The specimens were morphologically identified to species by the senior author (MAMS), an expert taxonomist in Neotropical anophelines^13^, and then were labeled and stored individually in silica gel at room temperature for subsequent analysis.

All methods were carried out in accordance with relevant guidelines and regulations. The research protocol regarding the use of HLC was approved by the Ethics Review Board of the University of São Paulo in June 2014 under approval number 159/14 expedited by the Department of Legal Medicine, Medical Ethics, Social and Occupational Medicine of the College of Medicine. All collectors were wearing clothing (trousers, socks, and long sleeved shirts) to protect themselves from direct contact with infectious bites in line with the current recommendation from the Ministry of Health^59^. Informed consent was obtained from all collectors.

### Laboratory processing samples and Plasmodium identification

Genomic DNA was extracted from each Anophelinae female collected in HLC, ST and BSS, and tested for the presence of *Plasmodium* species, following the protocol described in Sallum et al. 2019^15^.

## Data analysis

As each site was sampled only once, the sampling effort was able to detect the presence of *Plasmodium* in anophelines but not its absence. Thus, we performed data analysis for the positive sites (i.e., those that showed at least one *Plasmodium*-infected anopheline).

The first analysis identified the most plausible statistical distribution for describing and representing the variables of forest cover and deforestation timeline among sites. We divided the sites according to the presence of the following groupings: (1) all *Plasmodium*-infected anophelines; (2) *P*. *vivax*-*Ny*. *darlingi*; (3) *P*. *falciparum*-*Ny*. *darlingi*; and (4) *Plasmodium*-infected secondary vectors. Histogram plots were utilized to visualize the distribution of the variables. We adjusted the real data (i.e., the values of the forest cover and deforestation timeline variables) into a probability density function of Gaussian and log-normal distributions as follows:

$$g\left(x\right)= \frac{{e}^{-\frac{1}{2}(\frac{{(x-\mu )}^{2}}{{\sigma }^{2}})}}{\sigma \sqrt{2\pi }}$$

where the equation is the probability density function of the Gaussian distribution with two parameters: mean value (µ) and standard deviation (σ). This distribution is graphically represented by a symmetrical bell-shaped curve. We utilized the sample values (*x*) of forest cover and deforestation timeline to estimate these parameters (*µ* and *σ*) employing an algorithm of maximum likelihood estimation. Briefly, this algorithm is an optimizer for finding the minimum of the negative log-likelihood by obtaining the approximate covariance matrix and inverting the Hessian matrix at the optimum to accurately estimate the parameter values in R v. 3.6 (R Development Core Team; www.r-project.org)^60^.

$${f}_{x}\left(x\right)= \frac{1}{x}\frac{{e}^{(\frac{{(\mathrm{ln}(x)-\mu )}^{2}}{{\sigma }^{2}})}}{\sigma \sqrt{2\pi }}$$

is the probability density function when the logarithm of the random variable *X* is normally distributed:

$$\mathrm{ln}\left(X\right) \sim N(\mu ,\sigma )$$

In other words, *X* is log-normally distributed. The log-normal distribution can be interpreted as a more flexible version of the Gaussian distribution. Graphically, it can represent variable distributions that reach a peak (as a Gaussian distribution can) but with the possibility of showing the asymmetries across the space of observed data. A sudden peak with a smooth decline is a convex curve that is observed in the FMH^10,17^ and can be modeled and represented by a log-normal distribution. Hypothesis testing (Welch two-sample *t*-test) was applied to compare distributions (i.e., whether there were true differences in means not equal to 0) when a given variable showed a bimodal distribution. This was necessary to identify whether the bimodal distribution was truly bimodal or asymmetrically unimodal.

Poisson testing was performed to test whether there was a significant difference between the mean value of the number of *Plasmodium*-infected anophelines and its standard deviation. This variable (i.e., the number of *Plasmodium*-infected anophelines = 1, 2, 3 …31 per site) following a Poisson distribution, was the response variable. Forest cover was converted into accumulated deforestation (100%—forest cover). Pearson’s product-moment correlation was applied to test whether the true correlation between the accumulated deforestation and the deforestation timeline was not equal to 0. The accumulated deforestation and the deforestation timeline were categorized as binary variables (1, 0) according to^61^ and their mean values were used as a cut-off. Conceptual work by^61^ defined the limiting threshold of > 50% forest cover in tropical rainforests to allow for conservation of adequate gene flow to maintain natural populations in small and large patches. We used this value as a cut-off for classifying a site as preserved, or otherwise, as degraded. The accumulated deforestation baseline (0, preserved) and exposed (1, degraded) was based on the DMH, which states that increased deforestation leads to increased malaria^33,35^. The work by^17^ simulated the FMH using mathematical models and depicted malaria risk over periods longer than a decade; here we used 25 years as cut-off for classifying new or old settlements. The deforestation timeline conditions of baseline (0, old sites) and exposed (1, new sites) were based on the FMH, in which malarial risk is shown to increase in the early years of human settlement^10,29^. Combinations of these variables were applied to yield four specific explanatory variables: (1) new-preserved (NP), (2) new-degraded (ND), (3) old-preserved (OP), and (4) old-degraded (OD). It is expected that the new-degraded landscape scenarios have the highest malarial exposure risk. The ratio of mean numbers of *Plasmodium*-infected anophelines between a given landscape scenario and the baseline was calculated as a proxy to the malarial exposure risk^62^. The mean numbers of infected anophelines following a Poisson distribution were estimated by means of the general model equation as follows:

$$Poisson\left(\lambda \right)={exp}^{{\beta }_{0}+{\beta }_{n}{x}_{n}+ {\beta }_{n}{C}_{n}}$$

where *λ* is the estimation of the mean number of *Plasmodium*-infected anophelines in each site and *X* is the set of four explanatory variables (NP, ND, OP, OD) and *C* is the set of two control variables (number of collectors, period of collection being 6 h or 12 h).

A generalized linear model (GLM) approach was used to estimate the *Poisson* model coefficients (*β*_*n*_) ^63^. From the values of the *Poisson*-GLM coefficients, the incidence density ratio (IDR) of infected anophelines was estimated for the set of explanatory variables adjusted by control variables^62^. An adjusted IDR greater or less than 1 indicated malarial exposure risk or protection, respectively; otherwise, the landscape scenario had no clear effect on malarial exposure. All the tests performed had a significance threshold of 5%.

## Supplementary Information

Supplementary Information
